# Dietary Status and the Effect of Flaxseed Supplementation on the Severity of Perimenopausal Symptoms

**DOI:** 10.7759/cureus.79725

**Published:** 2025-02-26

**Authors:** Rashmi Shrivastava, Sandeep Bhattacharya, Narsingh Verma, Abbas A Mehdi, Amita Pandey, Jamal A Ansari

**Affiliations:** 1 Physiology, King George's Medical University, Lucknow, IND; 2 Physiology, Hind Institute of Medical Sciences, Lucknow, IND; 3 Biochemistry, Era's Lucknow Medical College and Hospital, Era University, Lucknow, IND; 4 Obstetrics and Gynecology, King George's Medical University, Lucknow, IND; 5 Chemistry, Integral University, Lucknow, IND

**Keywords:** dietary intake, nutrients, perimenopausal symptoms, relationship, severity

## Abstract

Background

Perimenopause is a transitional phase leading to menopause, increasing the risk of various associated diseases. Given the potential adverse effects of long-term conventional treatments, flaxseed serves as a promising nonpharmacological alternative for perimenopausal prevention.

Objective

Given the limited research on perimenopause and the potential of flaxseed as a prophylactic therapy for associated symptoms and their severity in later stages or before menopause, this study aims to examine the effects of flaxseed on perimenopausal symptom severity and its relationship with dietary nutrient intake.

Methods

Data from 145 participants in this randomized, single-blind, placebo-controlled trial were analyzed and compared before and after the intervention in two groups: Group A (flaxseed) and Group B (placebo). The study aimed to evaluate the impact of flaxseed on the severity of perimenopause-related symptoms and dietary nutrient intake while also assessing the relationship between symptom severity and dietary nutrients.

Results

The study’s findings reveal that flaxseed supplementation significantly reduced the severity of perimenopausal symptoms compared to the placebo (p < 0.001), with effect sizes ranging from -0.86 to -1.55. Among the nutrients analyzed - energy, protein, fat, carbohydrates, fiber, iron, and calcium - only energy, fiber, calcium, and iron showed significant changes from baseline to follow-up in the flaxseed group compared to the placebo. Additionally, significant correlations were observed between symptom severity and dietary nutrient intake. Specifically, sleep disturbances were significantly associated with energy intake (before intervention: r = 0.251, p = 0.024; after intervention: r = 0.265, p = 0.018), while vaginal dryness was significantly correlated with fiber intake (before intervention: r = 0.402, p = 0.001; after intervention: r = 0.267, p = 0.017).

Conclusions

Flaxseed may help alleviate certain perimenopausal symptoms, making it a useful supplemental nonpharmacological preventive therapy.

## Introduction

As women approach menopause, they often experience irregular menstruation and sporadic amenorrhea, marking the perimenopausal phase. During this transition, oocyte counts decline, follicle-stimulating hormone levels rise, and estrogen levels fluctuate unpredictably. Typically occurring between the ages of 45 and 55, this stage reflects the progressive decline in ovarian function over several years [1]. Perimenopause is often accompanied by a wide range of distressing physical symptoms that can significantly impact health and quality of life. Common symptoms include hot flashes, night sweats, mood swings, joint pain, insomnia, headaches, migraines, weight gain, and bone mineral density loss. Additionally, this phase can disrupt daily life and work, leading to psychological burdens for both those experiencing it and those around them. Furthermore, perimenopause is associated with an increased risk of conditions such as coronary artery disease, diabetes, hypertension, and insomnia [2].

A woman’s reproductive life necessitates distinct nutritional requirements compared to men due to the pronounced physical and hormonal changes occurring throughout different life stages. Despite the increasing number of women experiencing perimenopause and its associated symptoms, there remains a gap in knowledge regarding the role of diet and nutrition in alleviating symptom severity [3].

It is crucial to evaluate risk factors related to lifestyle choices and the level of awareness that can be modified to mitigate or even prevent menopausal symptoms. Nutrition, a key aspect of lifestyle, is often overlooked, particularly among women in rural areas. However, shifting lifestyle patterns - such as the widespread consumption of nutrient-poor, easily accessible fast food - have also led to an increased prevalence of diet-related diseases and perimenopause-associated symptoms among urban women. Recent findings suggest that dietary interventions, particularly those that increase fiber intake while reducing fat consumption, may help alleviate vasomotor symptoms in postmenopausal women [4].

The effects of phytoestrogens and phytosterols on human health have garnered increasing attention, as these compounds influence endocrine pathways and exhibit clinical properties similar to 17β-estradiol [5]. Herbal estrogens, such as phytoestrogenic agents, mimic the body’s natural estrogen production and may help mitigate menopausal symptoms. Phytoestrogens are found in fruits, vegetables, and grains, with flaxseed being the most common and abundant source. Studies suggest that within 12 weeks, flaxseed and other phytoestrogens can provide relief from menopausal symptoms [6].

Despite existing research, limited knowledge exists regarding the impact of flaxseed on menopausal symptom severity, particularly in relation to diet and nutrition. Given the need for further investigation, this study aims to assess the effect of flaxseed supplementation on perimenopausal symptom severity. Additionally, it explores the relationship between perimenopausal symptoms and dietary modifications over a three-month follow-up period.

## Materials and methods

This single-blind, randomized, placebo-controlled trial recruited 200 participants from the outpatient clinic of the Obstetrics and Gynecology Department at Queen Mary’s Hospital, King George’s Medical University, Lucknow, India. Of these, 12 individuals were excluded for other reasons, 13 declined participation, and 30 did not meet the inclusion criteria. Based on predefined inclusion and exclusion criteria, 145 eligible participants were randomly assigned to two groups using a computer-generated random number table. Group A (n = 72) received the flaxseed intervention, while Group B (n = 73) received a placebo. Participants were allocated using simple random sampling.

**Figure 1:**
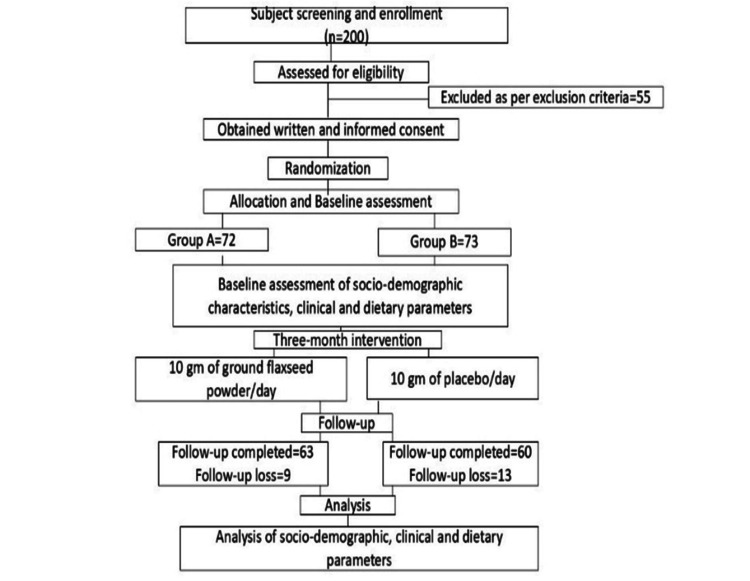
Study design

Following supplementation, 63 participants in Group A and 60 in Group B completed the study. Group A had a follow-up loss of nine participants, whereas Group B lost 13 participants. The sample size was determined based on prior research (Figure 1) [7].

Sociodemographic variables were evaluated at baseline for all participants. Clinical and dietary parameters were assessed both before and after the intervention to analyze changes over time.

Subjects

Participants aged 40 to 55 were recruited based on the International Classification of Diseases (ICD-10) code N-95.9 (unspecified menopausal and perimenopausal disorder) [8] and the study’s inclusion and exclusion criteria. Eligible participants had not taken supplements, standard pharmaceutical treatments (such as estrogen or phytoestrogens), or consumed foods rich in flavonoids or isoflavonoids for at least three months before or during the trial.

Exclusion criteria included surgical menopause, hysterectomy, abnormal ovarian or uterine anatomy, hormone replacement therapy, selective serotonin reuptake inhibitors, mental health conditions (such as depression), or thyroid disorders. Additionally, individuals with heart, liver, or kidney disease, those taking sedatives or anti-anxiety medications, smokers, and drug users were excluded.

Participants were diagnosed based on their medical history and clinical symptoms after meeting the inclusion and exclusion criteria. The study was ethically approved by the institutional ethics committee of King George’s Medical University, and informed consent was obtained from all participants.

To ensure single-blind conditions, participants in Group A received 10 g of flaxseed powder in an amber glass vial coded at the bottom, while Group B received identical vials containing roasted wheat flour. The unique codes on the vials were known only to the researchers and could not be interpreted by participants. Patients were instructed to store the supplement bottles in a cool, dry place and to consume only one bottle per day.

The flaxseed powder and whole wheat flour were supplied by Ceyon Healthcare Private Limited (Lucknow, India). Compliance was monitored through monthly bottle collection, weekly phone calls, routine emails, and participant logs. Additionally, participants were advised to maintain their usual diet and exercise routine throughout the study.

Clinical symptoms assessment

Clinical symptoms were evaluated using the Menopause Rating Scale (MRS) [9], which consists of 11 components rated on a scale from 0 (no complaints) to 4 (extremely severe symptoms). Each level of symptom severity was assigned a corresponding score. Participants indicated the severity of their symptoms by selecting one of five response options, reflecting their subjective assessment of each issue.

Dietary assessment

Dietary intake was assessed using a validated three-day, 24-hour dietary recall tool, adapted from the Food and Agriculture Organization of the United Nations’ resource guide for dietary assessment (Rome, 2018) [10]. This tool recorded meal names, meal timings, and detailed descriptions of all foods consumed over a 24-hour period for three days. Participants provided information on the types and quantities of food consumed at each meal, along with meal timing. The dietary recall was conducted under the supervision of the institute’s senior dietitian.

To analyze macronutrient and micronutrient intake, dietary data were evaluated using the Nutritive Value of Indian Foods by Gopalan [11], with oversight from an expert dietitian.

Statistical analysis

Data were presented as mean, SD, frequency, percentage, and number (n). Relationships between sociodemographic variables were analyzed using the chi-square test. The Kolmogorov-Smirnov test was applied to assess the normality of data distribution.

For within-group comparisons, the Wilcoxon matched pair test was used for nonparametric data, while the paired t-test was applied for parametric data. For between-group comparisons, parametric data were analyzed using the independent t-test, and nonparametric data were assessed using the Mann-Whitney U test.

Statistical significance was set at p < 0.05, with p < 0.01 and p < 0.001 considered moderately significant and highly significant, respectively. All statistical analyses were performed using IBM SPSS Statistics for Windows, Version 24.0 (Released 2016; IBM Corp., Armonk, NY, USA).

## Results

The ages of perimenopausal women were categorized into three groups, each spanning five years, ranging from 40 to 55 years in both study groups. In Group A, the majority of participants (55.6%) fell within the 40- to 45-year category, while in Group B, this age group also had the highest proportion of participants (53.3%) compared to the other two categories.

Regarding residential distribution, most participants in both groups resided in urban areas. In terms of BMI, a significant proportion of participants in both groups were classified as obese (BMI > 25), with 79.4% in Group A and 66.7% in Group B. In Group A, 1.6% of individuals had a BMI below 18, 6.3% fell within the 18-22.9 range, and 12.7% had a BMI of 23-24.9. In Group B, 1.7% had a BMI below 18, 10% were within the 18-22.9 range, and 21.7% had a BMI of 23-24.9.

A chi-square test revealed no significant differences between the groups for these variables at baseline (Table 1).

**Table 1:** Baseline sociodemographic characteristics for Group A and Group B Data are expressed in frequency (percentage), i.e., n (%). x = chi-square test, nsp > 0.05

Variables	Category	Group A, n (%)	Group B, n (%)	p-Value
Age	40-45	35 (55.6)	32 (53.3)	^x^t^ns^ = 0.450
	46-50	23 (36.5)	23 (38.3)	
	51-55	5 (7.9)	5 (8.3)	
Residential area	Village	12 (19)	11 (18.3)	^x^t^ns^ = 0.919
	City	51 (81)	49 (81.7)	
Physical activity	Sedentary	47 (74.6)	46 (76.7)	^x^t^ns^ = 0.797
	Moderate	13 (20.6)	14 (23.3)	
	Heavy	3 (4.8)	0	
Body build	Endomorphic	6 (9.5)	6 (10)	^x^t^ns^ = 0.784
	Mesomorphic	46 (13)	30 (50)	
	Ectomorphic	11 (7.5)	24 (40)	
BMI	<18	1 (1.6)	1 (1.7)	^x^t^ns^ = 0.452
	18-22.9	4 (6.3)	6 (10)	
	23-24.9	8 (12.7)	13 (21.7)	
	>25	50 (79.4)	40 (66.7)	

Frequency of symptoms in Group A and Group B before and after three-month intervention

Before the intervention, the most frequently reported symptoms in Group A were formication (49.2%), paresthesia (46%), vertigo (47.6%), fatigue (42.9%), and heart palpitations (41.3%). After the intervention, there was a shift, with more participants experiencing symptoms at mild to moderate levels, while those reporting severe to very severe symptoms decreased. This suggests an overall reduction in both the frequency and severity of menopausal symptoms in Group A after three months of intervention compared to baseline (Table 2).

**Table 2:** Frequency of symptoms in Group A (n = 63) Data are expressed in frequency (percentage), i.e., n (%).

Menopausal Symptoms	No complaint, n (%)		Light, n (%)		Medium, n (%)		Severe, n (%)		Very severe, n (%)	
	Before intervention	After three-month intervention	Before intervention	After three-month intervention	Before intervention	After three-month intervention	Before intervention	After three-month intervention	Before intervention	After three-month intervention
Sweating and hot flushes	0	9 (14.3)	2 (3.2)	25 (39.7)	13 (20.6)	28 (44.4)	25 (39.7)	1 (1.6)	23 (36.5)	0
Paresthesia	2 (3.2)	23 (36.5)	5 (7.9)	22 (34.9)	29 (46)	17 (27.0)	14 (22.2)	1 (1.6)	13 (20.6)	0
Insomnia	2 (3.2)	28 (44.4)	18 (28.6)	14 (22.2)	20 (31.7)	18 (28.6)	9 (14.3)	3 (4.8)	14 (22.2)	0
Nervousness	5 (7.9)	27 (42.9)	9 (14.3)	14 (22.2)	22 (34.9)	18 (28.6)	12 (19)	4 (6.3)	15 (23.8)	0
Melancholia	1 (1.6)	19 (30.2)	7 (11.1)	24 (38.1)	24 (38.1)	16 (25.4)	19 (30.2)	4 (6.3)	12 (19)	0
Vertigo	0	16 (25.4)	6 (9.5)	30 (47.6)	30 (47.6)	16 (25.4)	21 (33.3)	1 (1.6)	6 (9.5)	0
Fatigue	0	15 (23.8)	9 (14.3)	35 (55.6)	27 (42.9)	11 (17.5)	19 (30.2)	2 (3.2)	8 (12.7)	0
Arthralgia and myalgia	3 (4.8)	13 (20.6)	10 (15.9)	35 (55.6)	22 (34.9)	13 (20.6)	20 (31.7)	2 (3.2)	8 (12.7)	0
Headache	8 (12.7)	11 (17.5)	4 (6.3)	33 (52.5)	20 (31.7)	17 (27)	22 (34.9)	2 (3.2)	9 (14.3)	0
Heart palpitation	5 (7.9)	8 (12.7)	5 (7.9)	37 (58.7)	26 (41.3)	18 (28.6)	23 (36.5)	0	4 (6.3)	0
Formication	2 (3.2)	2 (3.2)	0	11 (17.5)	5 (7.9)	30 (47.6)	25 (39.7)	20 (31.7)	31 (49.2)	0

In Group B, the frequency of menopausal symptoms ranged from mild to very severe. The most commonly reported symptoms were formication (51.7%), vertigo (51.7%), fatigue (46.7%), heart palpitations (43.3%), paresthesia (41.7%), arthralgia/myalgia (41.7%), nervousness (40%), and melancholia (40%). Notably, the percentage of participants experiencing symptoms in the severe category increased, indicating a worsening of symptom frequency in Group B between baseline and the three-month follow-up (Table 3).

**Table 3:** Frequency of symptoms in Group B (n = 60) Data are expressed in frequency (percentage), i.e., n (%).

Menopausal symptoms	No complaint, n (%)		Light, n (%)		Medium, n (%)		Severe, n (%)		Very severe, n (%)	
	Before intervention	After three-month intervention	Before intervention	After three-month intervention	Before intervention	After three-month intervention	Before intervention	After three-month intervention	Before intervention	After three-month intervention
Sweating and hot flushes	0	2 (3.3)	3 (5.0)	19 (31.7)	18 (30.0)	17 (28.3)	19 (31.7)	20 (33.3)	20 (33.3)	2 (3.3)
Paresthesia	6 (10.0)	13 (21.7)	11 (18.3)	21 (35)	25 (41.7)	14 (23.3)	11 (18.3)	11 (18.3)	7 (11.7)	1 (1.7)
Insomnia	1 (1.7)	10 (16.7)	20 (33.3)	22 (36.7)	15 (25.0)	15 (25)	13 (21.7)	12 (20)	11 (18.3)	1 (1.7)
Nervousness	3 (5.0)	14 (23.3)	14 (23.3)	23 (38.3)	24 (40.0)	11 (18.3)	9 (15.0)	11 (18.3)	10 (16.7)	1 (1.7)
Melancholia	1 (1.7)	12 (20)	13 (21.7)	23 (38.3)	24 (40.0)	15 (25)	8 (13.3)	7 (11.7)	14 (23.3)	3 (5)
Vertigo	1 (1.7)	9 (15)	13 (21.7)	24 (40)	31 (51.7)	18 (30)	7 (11.7)	6 (10)	8 (13.3)	3 (5)
Fatigue	0	8 (13.3)	11 (18.3)	25 (41.7)	28 (46.7)	18 (30)	12 (20.0)	7 (11.7)	9 (15.0)	2 (3.3)
Arthralgia and myalgia	7 (11.7)	11 (18.3)	9 (15.0)	18 (30)	25 (41.7)	21 (35)	11 (18.3)	7 (11.7)	8 (13.3)	3 (5)
Headache	2 (3.3)	4 (6.7)	6 (10.0)	22 (36.7)	23 (38.3)	23 (38.3)	20 (33.3)	10 (16.7)	9 (15.0)	1 (1.7)
Heart palpitation	6 (10.0)	8 (13.3)	8 (13.3)	19 (31.7)	26 (43.3)	27 (45)	17 (28.3)	6 (10)	3 (5.0)	0
Formication	2 (3.3)	2 (3.3)	0	2 (3.3)	6 (10.0)	11 (18.3)	21 (35.0)	38 (63.3)	31 (51.7)	7 (11.7)

Impact on severity symptom score in Group A and Group B

All symptoms in Group A showed a significant reduction after three months, with most symptoms improving at p < 0.001. Notable improvements were observed in hot flushes, heart discomfort, sleep problems, depressive mood, irritability, anxiety, exhaustion, sexual problems, bladder issues, vaginal dryness, and joint/muscular pain.

In Group B, some symptoms also showed significant improvement, including hot flushes, heart discomfort, physical/mental exhaustion, bladder problems, and anxiety, but to a lesser extent. However, no significant improvement was observed in sleep problems, depressive mood, irritability, vaginal dryness, or joint/muscular pain. Additionally, sexual problems worsened in Group B after three months.

At baseline, there were no significant differences between the groups, indicating similar initial symptom severity. Overall, flaxseed supplementation in Group A led to significant reductions in menopausal symptoms, particularly hot flushes, heart discomfort, anxiety, and physical/mental exhaustion. In contrast, Group B (control) experienced minimal improvements, with some symptoms persisting or worsening. While between-group differences were not statistically significant, the within-group improvements highlight the potential benefits of flaxseed supplementation in managing perimenopausal symptoms (Table 4).

**Table 4:** Changes in MRS symptoms scores in Group A and Group B Values are expressed in mean ± SD or percentages. w = Wilcoxon matched pair test, u = Mann-Whitney U test, nsp > 0.05, *p < 0.05, **p < 0.01, *** p < 0.001 MRS, Menopause Rating Scale

Variables	Group A (n = 63)		Group B (n = 60)		p-Value		
	Before intervention	After three-month intervention	Before intervention	After three-month intervention	Within the group before intervention and after the three-month intervention		Group A vs. Group B at baseline
					Group A	Group B	
Hot flushes	3.09 ± 0.83	1.33 ± 0.74	2.93 ± 0.91	2.31 ± 0.96	^w ^p^***^ = <0.001	^w ^p^***^ = <0.001	^u^0.332^ns^
Heart discomfort	2.49 ± 1.01	0.93 ± 0.83	2.31 ± 0.98	1.93 ± 1.05	^w ^p^***^ = <0.001	^w ^p^**^ = 0.015	^u^0.387^ns^
Sleep problem	2.23 ± 1.18	0.93 ± 0.96	2.21 ± 1.15	2.20 ± 1.08	^w ^p^***^ = <0.001	^w ^p^ns^ = 0.782	^u^0.898^ns^
Depressive mood	2.36 ± 1.22	0.98 ± 0.99	2.15 ± 1.11	2.05 ± 0.96	^w ^p^***^ = <0.001	^w ^p^ns^ = 0.298	^u^0.255^ns^
Irritability	2.53 ± 0.98	1.07 ± 0.90	2.35 ± 1.11	2.38 ± 1.10	^w ^p^***^ = <0.001	^w ^p^ns^ = 0.755	^u^0.243^ns^
Anxiety	2.42 ± 079	1.03 ± 0.76	2.41 ± 0.97	2.20 ± 1.00	^w ^p^***^ = <0.001	^w ^p^*^ = 0.035	^u^0.708^ns^
Physical and mental exhaustion	2.41 ± 0.89	1.00 ± 0.74	2.31 ± 0.94	1.86 ± 1.15	^w^p^**^ = 0.001	^w ^p^***^ = <0.001	^u^0.473^ns^
Sexual problem	2.31 ± 1.04	1.06 ± 0.73	2.06 ± 1.16	2.38 ± 1.02	^w ^p^***^ = <0.001	^w ^p^*^ = 0.010	^u^0.205^ns^
Bladder problem	2.31 ± 1.18	1.15 ± 0.74	2.46 ± 0.98	1.91 ± 0.88	^w ^p^***^ = <0.001	^w ^p^***^ = <0.001	^u^0.709^ns^
Dryness of vagina	2.25 ± 0.98	1.15 ± 0.62	2.05±1.01	2.08±0.90	^w ^p^***^ = <0.001	^w ^p^ns^ = 0.679	^u^0.224^ns^
Joint and muscular pain	3.31 ± 0.87	2.07 ± 0.78	3.31 ± 0.91	3.41 ± 0.86	^w^p^**^ = 0.001	^w ^p^ns^ = 0.083	^u^0.907^ns^

Impact on nutritional status in both Group A and Group B before and after three-month intervention

Group A experienced significant reductions in total energy intake along with increases in calcium and iron intake. In contrast, Group B showed a significant increase in total energy intake, with no notable changes in other nutrients. Although between-group comparisons did not reveal statistically significant differences for any nutrient, these findings suggest that dietary changes were more pronounced in Group A over the three-month period. The study’s effect sizes ranged from -0.003 to 0.27 before the intervention and from -0.10 to 0.69 after the intervention (Table 5).

**Table 5:** Changes in nutritional status before and after three-month intervention in perimenopausal women (n = 123) Data are expressed as mean ± SD and percentages. T = independent t-test, t = paired t-test, U = Mann-Whitney U test, W = Wilcoxon test, p > 0.05 = no significant difference, **p < 0.01, *** p < 0.001

Nutrients	Group	Before intervention (mean ± SD)	After three-month intervention (mean ± SD)	Changes in percentage (%) before and after three-month intervention	p-Value	
					Within group before and after three-month intervention	Between groups before intervention
Total energy (Kcal)	Group A (n = 63)	2,124.60 ± 404.80	1,994.88 ± 372.20	-6.10%	^W^P^***^ <0.001	^U^P^ns^ = 0.812
	Group B (n = 60)	2,126.18 ± 452.43	2,236.01 ± 449.76	5.16%	^W^P^**^ = 0.006	
Protein (g)	Group A (n = 63)	77.03 ± 27.55	76.10 ± 26.38	-1.20%	^W^P^ns^ = 0.113	^U^P^ns^ = 0.146
	Group B (n = 60)	68.40 ± 17.06	68.44 ± 16.93	0.05%	^t^P^ns^ = 0.873	
Fat (g)	Group A (n = 63)	61.91 ± 33.43	60.48 ± 29.46	-2.30%	^W^P^ns^ = 0.612	^U^P^ns^=0.252
	Group B (n = 60)	52.79 ± 16.11	52.37 ± 14.85	-0.11%	^W^P^ns^ = 0.164	
Carbohydrate (g)	Group A (n = 63)	314.81 ± 98.61	312.90 ± 77.23	-0.60%	^t^P^ns^ = 0.980	^T^P^ns ^=0.977
	Group B (n = 60)	314.35 ± 79.91	321.04 ± 84.16	2.12%	^t^P^ns^ = 0.104	
Fiber (g)	Group A (n = 63)	5.36 ± 1.43	6.12 ± 1.42	0.12%	^t^P^***^ <0.001	^T^P^ns ^= 0.385
	Group B (n = 60)	5.13 ± 1.50	5.13 ± 1.54	0	^t^P^ns^ = 0.936	
Calcium (mg)	Group A (n = 63)	161.43 ± 60.13	183.70 ± 60.83	13.79%	^W^P^**^ <0.01	^U^P^ns^ = 0.796
	Group B (n = 60)	158.86 ± 59.16	158.54 ± 58.78	-0.20%	^W^P^ns^ = 0.279	
Iron (mg)	Group A (n = 63)	9.03 ± 1.91	10.19 ± 2.28	12.84%	^t^P^***^ <0.001	^T^P^ns^ = 0.528
	Group B (n = 60)	8.81 ± 2.01	8.78 ± 1.97	-0.34%	^t^P^ns^ = 0.645	

Association between symptom scores and nutrient intake in Group A and Group B before and after the three-month intervention

In Group A, a weak but significant positive correlation was observed between energy intake and sleep problems at baseline (r = 0.251, p = 0.024), which remained significant after the follow-up (r = 0.265, p = 0.018). This suggests that energy intake may influence sleep issues. In contrast, no significant association was found between energy intake and sleep problems in Group B at either time point.

Regarding heart discomfort and fat intake, no significant correlation was found at baseline in Group A. However, after the follow-up, a weak positive correlation emerged, approaching significance. In Group B, a weak negative correlation at baseline was significant at the 5% level (r = -0.239, p = 0.033), which strengthened significantly after the follow-up (r = -0.432, p < 0.001).

A moderate positive correlation was found between bladder problems and iron intake in Group A at baseline, significant at the 1% level. This correlation strengthened and remained significant after the follow-up. However, no noteworthy correlation was found at either time point in Group B.

For vaginal dryness and fiber intake, a highly significant moderate positive correlation was observed at baseline (r = 0.402, p = 0.001), which weakened slightly but remained significant after the three-month follow-up (r = 0.267, p = 0.017) in Group A. No significant correlation was found in Group B at either time point.

Additionally, a weak negative correlation was observed in Group B between depressive mood and fat intake at both baseline (p = 0.462) and follow-up (p = 0.430), as well as between bladder problems and fat intake at baseline (p = 0.437) and follow-up (p = 0.343). However, no significant correlations were found for these symptoms in Group A.

The remaining symptoms showed no significant associations with nutrient intake at either time point in either group. Only the significant correlations are presented in Table 6, as other symptoms did not exhibit meaningful relationships.

**Table 6:** Correlation of individual symptom score with dietary intake of nutrients in Group A (n = 63) and Group B (n = 60) Spearman correlation analysis was performed. * Correlation was significant at p< 0.05 (one tailed). ** Correlation was significant at p < 0.01 (one tailed).

Symptoms	Dietary nutrient intake from diet	Symptoms correlation with dietary nutrient intake in groups (one tailed)			
		Group A		Group B	
		Before intervention	After three-month intervention	Before intervention	After three-month intervention
Sleep problem	Energy	0.251^*^ p = 0.024	0.265^*^ p = 0.018	-0.085 p = 0.259	-0.132 p = 0.158
Heart discomfort	Fat	0.126 p = 0.163	0.171^*^ p = 0.090	-0.239^*^ p = 0.033	-0.432^**^ p = 0.000
Depressive mood	Fat	0.012 p = 0.462	0.023 p = 0.430	-0.303^**^ p = 0.009	-0.301^**^ p = 0.010
Bladder problem	Fat	0.020 p = 0.437	0.052 p = 0.343	-0.232^*^ p = 0.044	-0.368^**^ p = 0.002
Bladder problem	Iron	0.297^**^ p = 0.009	0.339^*^ p = 0.003	0.085 p = 0.259	-0.013 p = 0.460
Dryness of vagina	Fiber	0.402^**^ p = 0.001	0.267^**^ p = 0.017	0.072 p = 0.293	0.057 p = 0.333

Thus, in Group A, correlations tend to become stronger or reach significance after the intervention, suggesting that flaxseed supplementation may have a beneficial impact on symptoms influenced by nutrient intake. In contrast, Group B exhibits limited or negative correlations following the intervention, emphasizing a differential effect of flaxseed compared to the placebo. In the nutrient-symptom relationships observed, energy intake is related to sleep problems, fat intake is associated with heart discomfort and depressive mood, iron intake correlates with bladder problems, and fiber intake is strongly linked to vaginal dryness. This analysis highlights the nuanced role of dietary nutrients and suggests that flaxseed may modulate their effects on perimenopausal symptoms.

## Discussion

The present investigation examines the effects of a 10 g flaxseed intervention on the intensity of perimenopausal symptoms and its correlation with dietary nutrient consumption. Dietary phytoestrogens can influence menopausal symptoms, as lignans present in plant foods exhibit estrogenic properties. Consequently, dietary habits may help mitigate estrogen deficiency during menopause [12].

Studies have demonstrated the significant impact of flaxseed on menopausal symptoms before and after data collection. Results indicate that symptoms such as vaginal dryness, irritation, depression, and anxiety were not significantly affected (p > 0.05). However, hot flashes, sleep disturbances, depression, and physical and mental fatigue showed significant differences between the flaxseed and placebo groups post-intervention [13]. A daily intake of 1,000 mg of flaxseed for six weeks significantly reduced the frequency and duration of menopausal symptoms, including hot flashes and night sweats [14]. Another study found that consuming flaxseed supplements for three months significantly alleviated menopausal symptoms in the intervention group (p < 0.001) [15]. Additionally, research on postmenopausal women revealed that flaxseed significantly reduced symptoms compared to the control group, with effects comparable to hormone replacement therapy [16].

Further studies support the role of flaxseed in symptom relief. Both flaxseed and wheat germ were found to significantly decrease (p < 0.0001) the severity of menopausal symptoms, though no statistically significant difference was observed between the two groups [17]. A supplement containing soy flour and flaxseed, rich in protein, total phenols, fiber, and minerals, significantly reduced MRS scores, central adiposity, and blood pressure [18]. Women in the intervention group who consumed 200 ml of soy milk experienced a substantial decrease in all Menopause-Specific Quality of Life (MENQOL) categories (p < 0.001) [19]. Similarly, those who consumed 200 ml of homemade soy milk for six weeks exhibited significantly lower MENQOL scores across all dimensions (p < 0.001) [20].

Depressive symptoms, measured by the overall mean BDI-II scores, showed a steady decline over time. In postmenopausal women with minimal to moderate depression, symptom severity decreased following phytoestrogen consumption [21]. During a soy-rich dietary phase, genistein and daidzein serum levels significantly increased, confirming dietary compliance. Conversely, after a 12-week soy-free diet, symptoms such as vaginal dryness and urge incontinence worsened significantly, while no other urogenital symptoms changed during either period [22]. Adjusted ORs were statistically significant for certain quartiles, but no consistent monotonic relationships were observed between dietary phytoestrogens or fiber and incident vasomotor symptoms [23].

A study involving 78 women in the experimental group and 36 in the control group examined the effects of a phytoestrogen-rich diet (flaxseed, soy) over 12 weeks. After three months, the intervention group had significantly lower scores for vaginal dryness, hot flashes, and overall menopausal symptoms, whereas the control group exhibited minimal changes [24]. Another randomized controlled study assigned 28 women to a flaxseed group, 31 to a soy flour group, and 28 to a wheat flour group, consuming their respective foods for four months. Those who consumed flaxseed experienced less intense hot flashes compared to the wheat flour group [25].

The findings of the current study align with previous research. Participants who received a 10 g flaxseed intervention for up to three months reported fewer perimenopausal symptoms than the placebo group. In Group A (flaxseed group), most subjects initially experienced moderate to severe symptoms, which improved to mild or no symptoms after the intervention. In contrast, Group B (placebo group) exhibited no notable changes post-intervention.

Analysis of symptom severity using MRS scores revealed a significant decrease in symptom scores in Group A compared to Group B. The study’s effect size ranged from -0.86 to -1.55. Examining dietary parameters, a study found significant differences in carbohydrate, fat, and fiber intake between the flaxseed and wheat germ placebo groups after a yearlong intervention in healthy menopausal individuals. However, macronutrient and energy intake remained consistent between the groups before and after consuming two slices of partially defatted flaxseed bread [26]. Research also suggests that flaxseed fiber, whether in tablet or powder form, enhances satiety and reduces energy consumption at subsequent meals [27].

According to the National Institute of Nutrition’s 2024 recommendations, participants had deficiencies in iron, calcium, and fiber, while their daily intake of energy, fat, and protein exceeded the Recommended Dietary Allowance [28]. After the flaxseed intervention, macronutrient intake, including energy and carbohydrates, changed significantly (p < 0.001). Similarly, micronutrients such as fiber, calcium, and iron also showed significant changes (p < 0.001) in the flaxseed group compared to the placebo. However, protein and fat intake remained unchanged before and after the intervention.

Statistically significant correlations between symptom scores and dietary intake of nutrients were observed in both groups. In Group A, a weak but significant association was found between dietary intake and symptom relief, except for depressed mood and fat-related bladder issues. Group B exhibited negative associations before and after treatment, though these associations were not statistically significant for heart pain, bladder issues, and depression. Overall, the findings suggest that flaxseed consumption may influence symptoms such as heart discomfort, bladder issues, sleep disturbances, and vaginal dryness by affecting dietary intake of energy, fat, iron, and fiber, highlighting the potential benefits of flaxseed beyond placebo effects.

Despite demonstrating the beneficial effects of flaxseed on perimenopausal symptoms and dietary status, this study has some limitations. A larger sample size and a longer study duration could enhance the reliability of results, while a double-blind study design could strengthen the findings. Additionally, as this study was conducted in a North Indian population, further research with diverse populations and varied study designs is needed for more comprehensive conclusions.

## Conclusions

Flaxseed reduces the intensity of menopausal symptoms and significantly influences the dietary intake of certain nutrients. When analyzing the relationship between nutrients and symptoms, only a few nutrients showed a significant correlation between the flaxseed and placebo groups before and after the intervention. This finding opens new avenues for research to explore the complex interplay between symptom severity and nutrient intake, potentially enhancing our understanding of disease pathophysiology. Such insights could contribute to the development of dietary intervention programs aimed at improving the quality of life for perimenopausal women.
